# Emotion and Anticipation in an Enactive Framework for Cognition (Response to Andy Clark)

**DOI:** 10.3389/fpsyg.2012.00398

**Published:** 2012-10-09

**Authors:** Etienne B. Roesch, Slawomir J. Nasuto, J. Mark Bishop

**Affiliations:** ^1^School of Systems Engineering, University of ReadingReading, UK; ^2^Department of Computing, Goldsmiths University LondonLondon, UK

Undeniably, anticipation plays a crucial role in cognition. By what means, to what extent, and what it achieves remain open questions. In a recent BBS target article, Andy Clark depicts an integrative model of the brain that builds on hierarchical Bayesian models of neural processing (Rao and Ballard, 1999; Friston, 2005; Brown et al., 2011), and their most recent formulation using the free-energy principle borrowed from thermodynamics (Feldman and Friston, 2010; Friston, 2010; Friston et al., 2010). Hierarchical generative models of cognition, such as those described by Clark, presuppose the manipulation of representations and internal models of the world, in as much detail as is perceptually available. Perhaps surprisingly, Clark acknowledges the existence of a “virtual version of the sensory data” (p. 4), but with no reference to some of the historical debates that shaped cognitive science, related to the storage, manipulation, and retrieval of representations in a cognitive system (Shanahan, 1997), or accounting for the emergence of intentionality within such a system (Searle, 1980; Preston and Bishop, 2002). Instead of demonstrating how this Bayesian framework responds to these foundational questions, Clark describes the structure and the functional properties of an action-oriented, multi-level system that is meant to combine perception, learning, and experience (Niedenthal, 2007).

As pointed out by Clark, extreme models within this framework reduce experience to a mere by-product of the relationship between neural anticipatory signal and motor commands. Rightfully, Clark is uncertain of the radical proposal that we might “do away with the need to appeal to goals and rewards” (p. 59), and attempts to reinstate some aspects of emotional experience in the form of a frame of reference against which is construed this action-oriented predictive framework. We submit that this argument falls short of momentum in accounting for a rich phenomenology. Emotional experience simply cannot be reduced to a frame of reference: embodiment and embeddedness are at the core of the organism’s identity in its lived world, and fundamental aspects of emotional experience (Niedenthal, 2007). These features of experience situate the organism in the environment, which perceives and interacts with its immediate surrounding. This situatedness can only arise from concentric cycles of operations that combine integrated levels of anticipation with enactive processes of interaction with the environment. Anticipation not only takes place in the brain, which is the preferred level of perspective in the models introduced by Clark, but also at the many levels contained within the body, and between the body and the environment (Kurthen, 2007). In such a framework, the brain is a necessary but not sufficient part of the enactive organism, and emotion takes a central place as the scaffolding to awareness, especially in action-oriented perspectives (Frijda et al., 1989).

By enactive processes, we refer to the closed-loop operations that settle the organism in the environment, which are grounded in, and shaped by the interaction itself, and that lead to the organism acting in ways optimal for adaptation and survival, supporting perception, learning, and experience. Within this framework, anticipation evidently plays a critical role in mitigating the interface with the environment. This situation relies on both the existence of natural constraints in the environment, and on the organism’s sensitivity to these constraints, which can either be based on their explicit description (i.e., a model of the world), or on the implicit, natural relationships that exist within the unitary system composed of both the organism and the environment. In the former case, prediction of future events occurs via the explicit manipulation of the description of the world against some metric of time. In contrast to this “weak” form of anticipation, which requires an expensive degree of energy to be sustained over time, a systemic form of anticipation may arise from the natural dispositions of both components of the system: a so-called “strong” anticipation based on the delayed feedback between the physical elements of the system, the properties of their synchronization and the strength of the coupling between them (Stepp and Turvey, 2010). The example of the oil drop in the saline solution that successfully exits a maze by following an appropriate Ph gradient illustrates this latter situation (Lagzi et al., 2010; http://www.youtube.com/watch?v=RXgP8rq_wfA). Arguably the oil drop does not possess or manipulate a model of the world to achieve this feat, but the interaction over time of the Ph of the oil drop with that of the saline solution leads the oil drop to move in the appropriate direction.

In this context, enactive processes contrast sharply with the models described by Clark in that they assume an implicit, cheaper (energy-wise), representation-lean reference to the future, as the natural, bidirectional relation between the organism and the environment unfolds over time. We thus submit that these models may be more immune to most informational bottlenecks evident in light of the requirements for surviving in the world, as exemplified by the historical debates we referred to earlier, and may therefore be more adequate than other models to account for cognition. At the level of the (embodied) brain, enactive processes may interface with the environment through several mechanisms including the synchronization of neural assemblies and large-scale integration of information (Engel et al., 2001; Varela et al., 2001). Much is needed to characterize these mechanisms.

In conclusion, we would like to formulate a word of caution. It is a mistake to conclude that, because model x can account for data y (epistemic concerns), therefore it must provide an accurate description of the inner workings of the brain (ontological description). Unless the model describes all the complexities of the embodied brain, embedded in the environment, one is almost always going to be making a conflation mistake. An extreme example might be representing “emotion” using a real number and, because this model can account for some data, wrongfully conclude that there must be an equivalent to the real number in the brain. Arguably, Clark’s review of a wide-range of data to justify hierarchical generative models falls into this category.
